# Preclinical safety testing and initial experience of a morcellation bag with four sealable ports

**DOI:** 10.1038/s41598-021-99934-1

**Published:** 2021-10-22

**Authors:** Michael Anapolski, Anja Schellenberger, Ibrahim Alkatout, Dimitrios Panayotopoulos, Alexander Gut, Stefan Soltesz, Sven Schiermeier, Thomas Papathemelis, Günter K. Noé

**Affiliations:** 1 Department of Obstetrics and Gynecology, Community Hospital Dormagen, University of Witten-Herdecke, Dr.-Geldmacher-St. 20, 41540 Dormagen, Germany; 2 Department of Obstetrics and Gynecology, Klinikum Ansbach, Ansbach, Germany; 3 Department of Obstetrics and Gynecology, University Hospitals Schleswig-Holstein, Campus Kiel, Kiel, Germany; 4 Department of Obstetrics and Gynecology, Community Hospital Dormagen, Dormagen, Germany; 5 Department of Obstetrics and Gynecology, Community Hospital Grevenbroich, Grevenbroich, Germany; 6 Department of Anesthesiology, Community Hospital Dormagen, Dormagen, Germany; 7 Department of Obstetrics and Gynecology, University Witten-Herdecke, Witten, Germany; 8 Department of Obstetrics and Gynecology, St. Marien Hospital Amberg, Amberg, Germany

**Keywords:** Medical research, Oncology, Risk factors

## Abstract

Electromechanical morcellation—so called power morcellation—is a minimally invasive approach to remove bulky lesions such as uterine fibroids. The spread of benign and malignant tissue due to morcellation is a major concern that might limit the use of laparoscopic interventions. We present an in vitro evaluation of the safety characteristics of a four-port endobag with closable trocar sleeves, and describe physical properties of the bag that may or may not allow passage through the hole. In addition, we report our preliminary experience of this tool when used for laparoscopic supracervical hysterectomies. The behavior of the endobag during the extraction process was analyzed by extracting opened and re-sealed bags filled with 20 ml blue dye solution through a wooden template, with incisions measuring 10 to 24 mm. The endobag was used in 50 subtotal hysterectomies during the morcellation procedure. In the in vitro test, no dye loss was recorded for incisions measuring 11–24 mm. The mean force required to pull the bag through the template was inversely proportional to incision size. No bag rupture occurred during the surgical procedures. The mean time taken to prepare the bag for morcellation was 7.1 min (range, 4–14 min), the mean duration of subtotal hysterectomy was 53.4 min (range, 20–194 min). The mean weight of the removed body of the uterus was 113.8 g (range, 13–896 g), the mean weight of tissue and fluid remaining in the bag after morcellation 7.9 g (range, 0–39 g). In the in vitro setting, the improved endobag signifies greater patient safety during bag extraction, along with less tissue traumatization due to a smaller incision in the abdominal wall. The improved ergonomic features of the bag permit the insertion of three trocars in the lower abdomen and avoid closure of unused access ports. Our preliminary experience has shown that the device can be used under routine conditions. Failure rates will be evaluated in future studies.

## Introduction

Modern laparoscopy, established in the 1980s, dispenses with the use of laparotomy for an increasing number of medical conditions^1^. Since the introduction of power morcellation in the early 1990s, it became feasible to remove large uterine fibroids and perform total or subtotal laparoscopic hysterectomies^2^. Thus, patients could benefit from the advantages of minimally invasive surgery.

In most modern operating rooms, electromechanical or so-called power morcellation involves the use of a round rotating blade that permits the extraction of tissue fragments without performing a laparotomy. However, the development of this powerful tool has been accompanied by critical warnings of its potential complications. Three types of complications or difficulties have been associated with the use of power morcellation: the risk of organ injury, difficulties in the identification of histopathological tissue patterns, and the risk of benign or malignant tissue spread in the abdomen. The likelihood of organ damage has been evaluated over the past decades. Minor and major complications, such as injury to abdominal organs have been described in the published literature as well as in complication databases^3,4^. Although these events must be taken into account, their overall risk in relation to the total number of morcellations carried out so far is low. The benefits of avoiding a laparotomy outweigh the disadvantages of laparoscopy. The experience and know-how gained by laparoscopic surgeons in the last few years have markedly reduced the risks associated with morcellation.

As far as the histopathological evaluation of tissue is concerned, the introduction of power morcellation necessitated the adjustment of clinical routine for the assessment of small tissue fragments^5^.

A growing body of evidence over the last few years has disclosed the sequelae of unprotected intraabdominal morcellation of uterine tissue, facilitating the spread of both benign and malignant tissue within the abdominal cavity^6–11^. Especially for uterine leiomyosarcoma (LMS), which is a rare uterine tumor, some authors have suggested an association between laparoscopic power morcellation and an increased rate of abdomino-pelvic dissemination, as well as adverse effects on disease-free survival and overall survival^12–15^. However, we have limited data on the subject and the above mentioned suggestions were not supported by a recent meta-analysis^16^. Although the prevalence of endometrial cancer is much higher than that of various subtypes of sarcoma, the likelihood of accidental morcellation is relatively low because histological sampling is performed prior to hysterectomy.

A number of endobag devices have been described in the published literature; their purpose is to reduce the risk of tissue and fluid dissemination during the morcellation process^17–22^.

Whereas the large majority of publications address the issue of morcellation in the narrow sense of shredding or comminution of tissue, some authors identified the extraction of the endobag from the abdominal cavity as a vulnerable step of the intervention. Traction forces acting on the bag raise the likelihood of losing its contents^20,21^.

The purpose of the present study was to determine a suitable mode of contained tissue morcellation that would permit safe bag retrieval after the procedure without enlargement of laparoscopic incisions and thus without additional traumatization of the abdominal wall. The large majority of solutions presented in the past allow the insertion of one or (maximum) two trocars into the bag via prefabricated openings. Another objective of the investigation was to establish a functional device that would enable the surgeon to use a maximum of three trocar ports during morcellation.

We present a polyurethane endobag with three sealed and re-sealable port sleeves in the lower abdomen, allowing the insertion of three instruments in addition to the umbilical trocar.

Based on a previously published study^21^, the safety properties of the new endobag were tested in the phase of sample removal from the abdominal cavity. Extraction of the endobag was first carried out in the in vitro setting. We also report preliminary use of the bag in 50 morcellation procedures.

## Methods

### In vitro test: bag extraction

**Figure 1:**
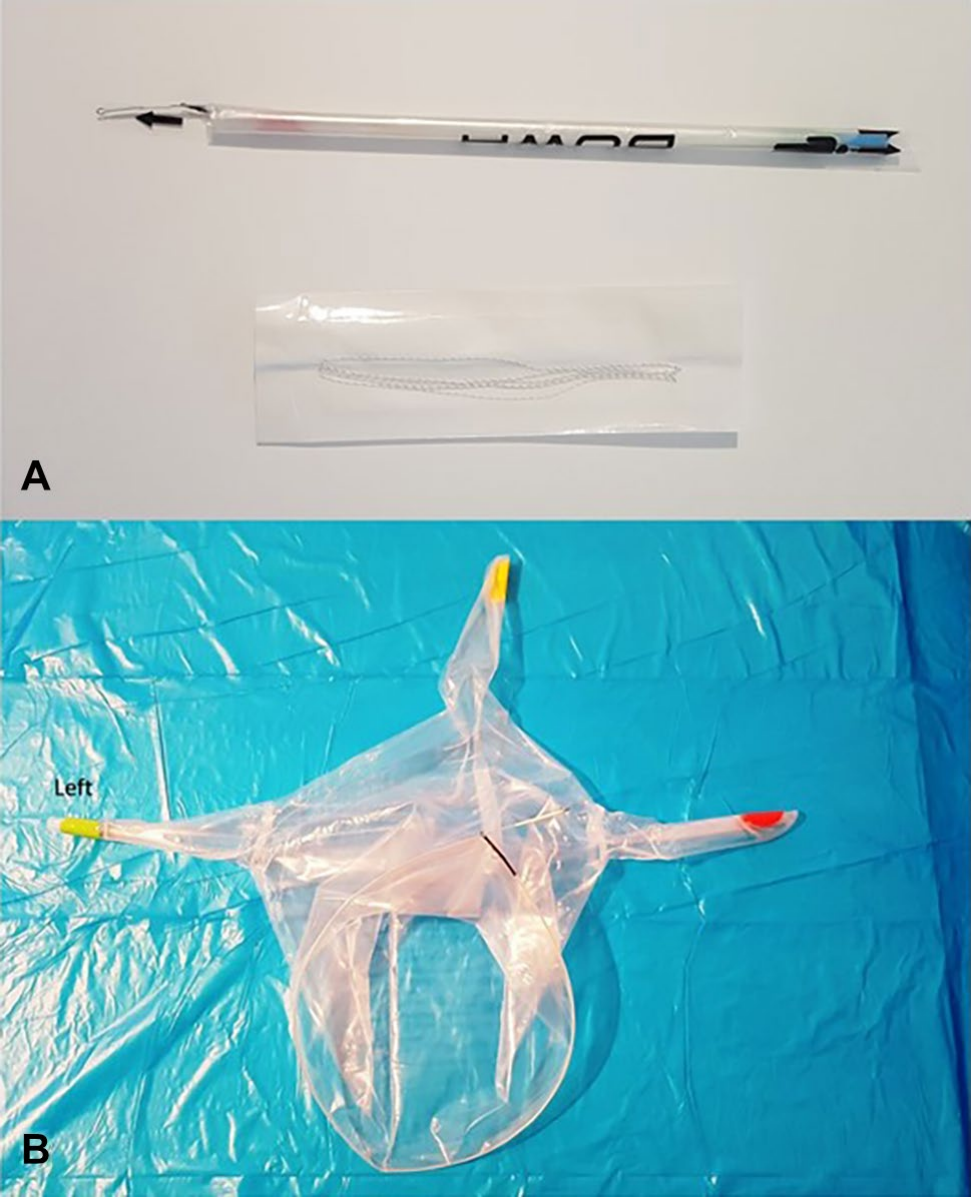
(**A**) Endobag with introducer and closure strings; (**B**) Unfolded endobag.

We used a new polyurethane bag for contained power morcellation (MetraBag, BOWA-Electronic GmbH, Gomaringen, Germany). The device is CE certified for controlled tissue morcellation in an enclosed system. According to the manufacturer’s specifications, the total volume of the bag is 3.5 l. In addition to the mouth of the bag (widest opening), the device offers three integrated sealed sleeves (Fig. 1). Depending on the surgeon’s approach and the patient’s anatomy (such as the size of the uterus and potential obesity), the bag permits the insertion of three trocars in the lower abdomen in addition to the umbilical trocar. Since all three sleeves are sealed by the manufacturer, the decision to use one, two, or three port sleeves can be made individually. For example, for large uteri it may be meaningful to use two or even all three sleeves to insert graspers along with the morcellator. In the present study, we used two of three ports in the lower abdomen. The sleeve ports should be extracted from the abdomen and opened outside the abdominal cavity in order to insert a morcellator or any other instrument. The trocar sleeves were re-sealed with closure strings after the end of the morcellation procedure and prior to bag extraction. Only the sleeves opened for the morcellator and/or instrumental trocars had to be closed at the end of surgery.

Based on previous data, we assumed that the device would be able to withstand a CO_2_ insufflation pressure of 12 mmHg^21^. Compared to the endobag described in the past^20,21^, the trocar sleeves reduce the risk of contact between the contents of the bag and the abdominal cavity/wall.

**Figure 2:**
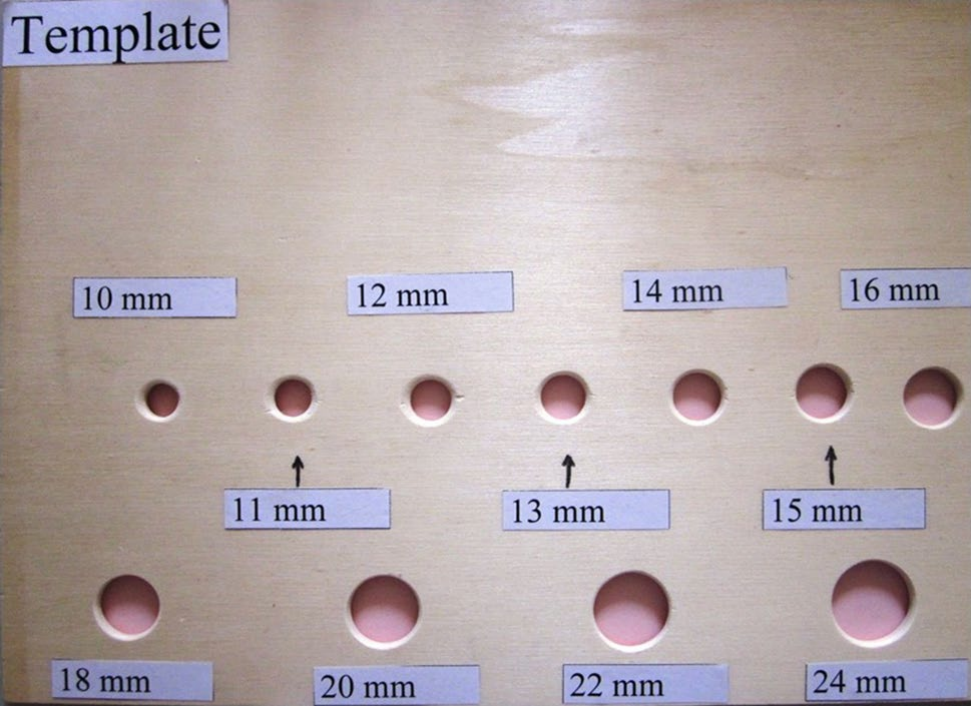
Wooden template.

**Figure 3:**
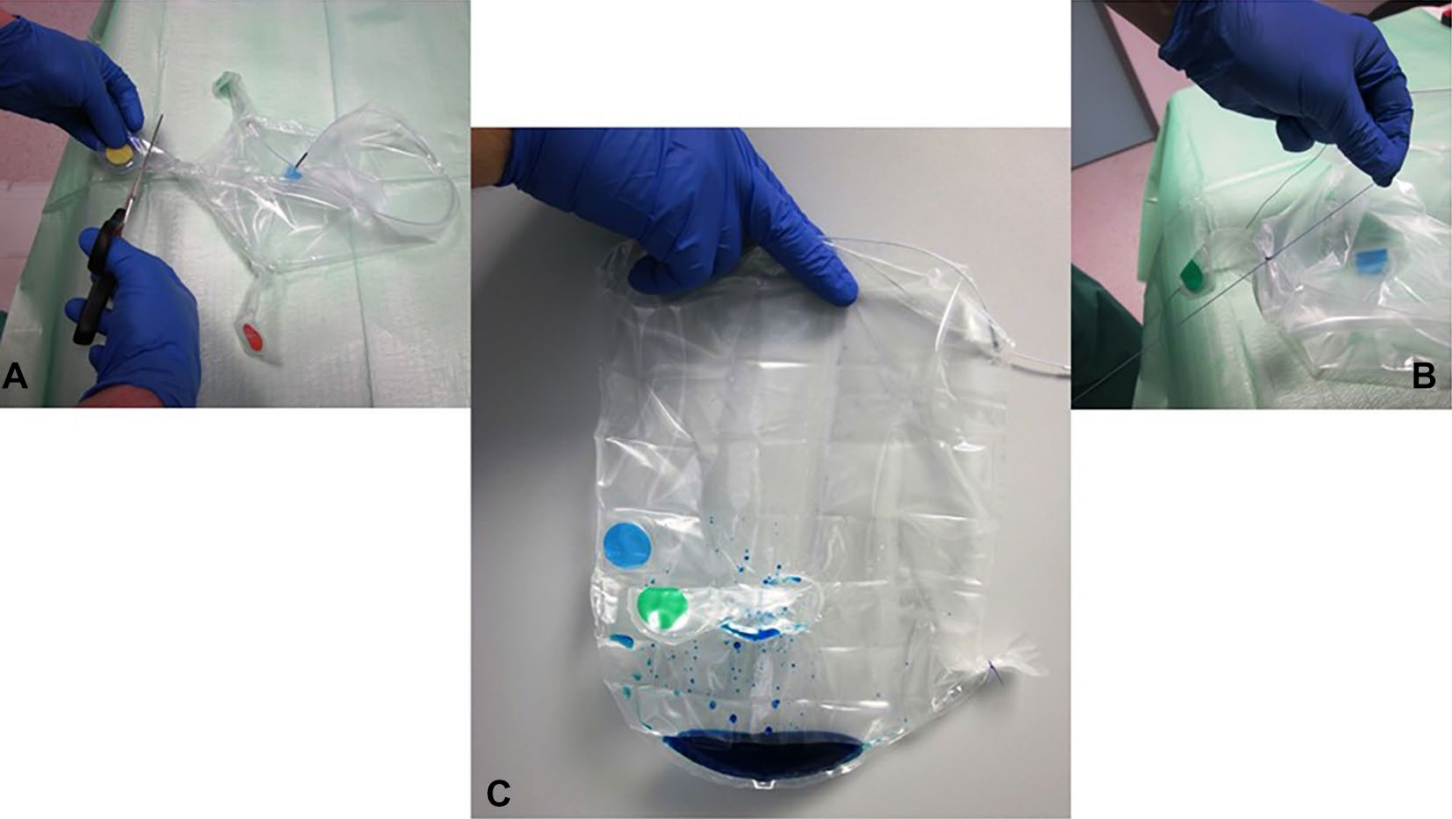
(**A**) Incision of the trocar sleeve; (**B**) Trocar sleeve is being re-sealed; (**C**) Blue dye inside the endobag.

**Figure 4:**
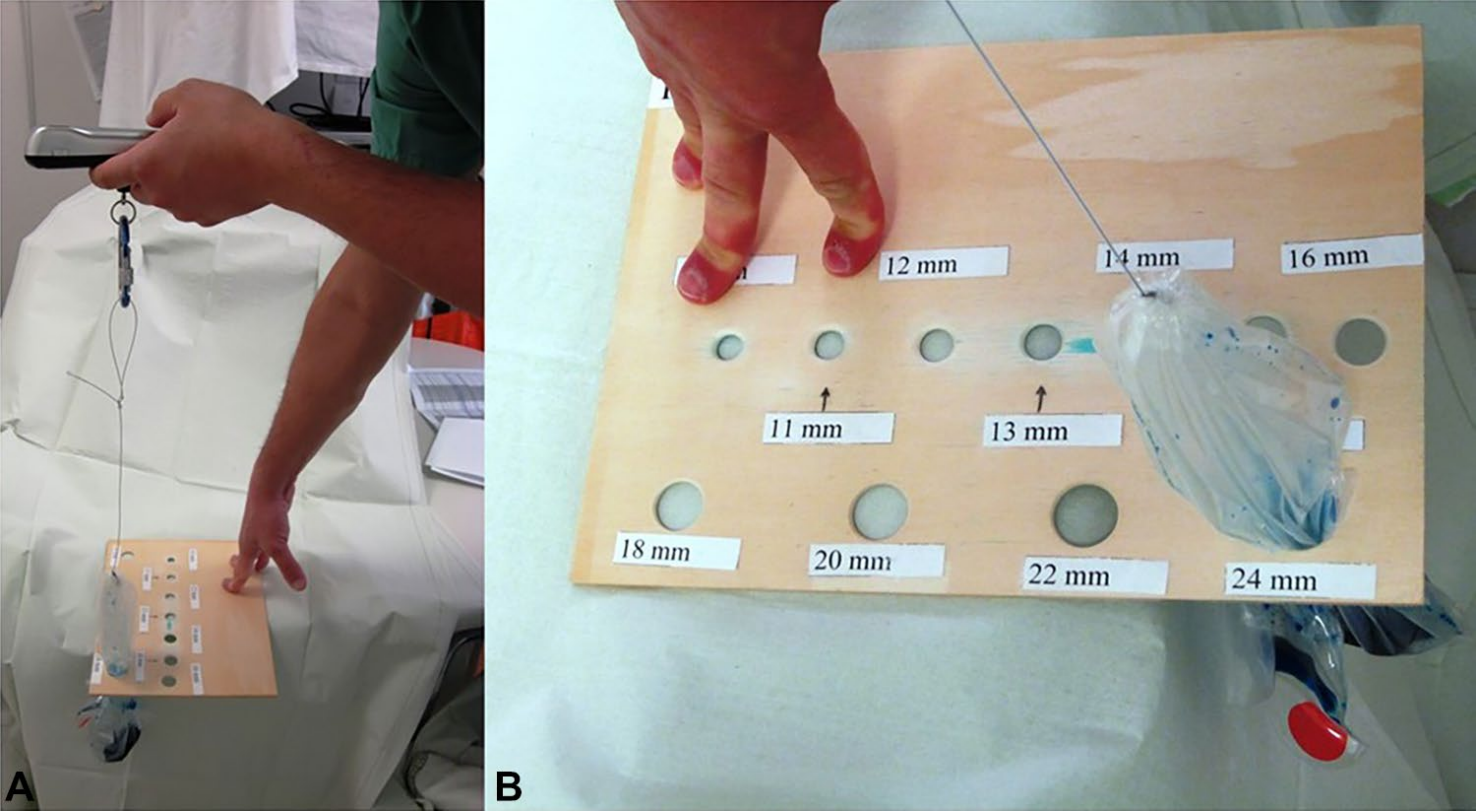
(**A**) and (**B**) Extraction of the endobag through the wooden template.

A wooden template with boreholes of 10 to 24 mm was used, as described earlier (Fig. 2). In order to simulate intraoperative conditions, two of three trocar sleeves were incised and then re-sealed with closure strings provided by the manufacturer. The bag was then filled with 20 ml Patent Blue V solution (Fig. 3). The quantity of the fluid was based on a pilot study that reported a mean residual volume of 12.1 ml in the endobag after morcellation^20^. White paper tissue was placed around the bag and template to register remote fluid loss during the extraction procedure. Direct fluid loss was registered at the edges of the boreholes of the wooden templates, as described earlier^21^. A handheld scale (Tschibo GmbH, Hamburg, Germany) was attached to the closure string of the bag mouth to measure the force applied during the extraction (Fig. 4). The following formula was used to calculate force: F = m * g (gravitational acceleration g = 9.81 N/kg). Ten extraction tests through the wooden template were performed for each borehole, and fluid loss—either direct or remote—was registered.

### Application of the endobag during power morcellation

The study was reviewed and approved by the ethics committee of the University of Witten-Herdecke. The research work was performed in accordance with relevant guidelines and regulations. Informed consent was obtained from all subjects. All patients had been referred to our center for laparoscopic procedures with preservation of the cervix.

Subtotal hysterectomy was performed in two groups of patients: (a) symptomatic pre- and perimenopausal patients referred to our center due to leiomyoma and/or hypermenorrhea, and (b) patients scheduled to undergo pelvic floor surgery in case a subtotal hysterectomy was part of the planned procedure. In all cases we used two instrumental ports: one in the left lower abdomen and the other in the suprapubic area. After subtotal hysterectomy, the body of the uterus was placed in the right upper abdomen. In-bag morcellation was the last step of the procedure. The endobag was introduced into the abdomen via the umbilical port and unfolded in the abdominal cavity (Video 1). The uterus and the fallopian tubes—in case the patient had consented to a salpingectomy—were placed inside the bag. The mouth of the bag was closed by applying traction to the closure string; the tip of the string was then removed from the abdomen via the umbilical trocar. The umbilical trocar was reinserted into the abdomen bluntly. The left and central lower abdominal ports were extracted via the abdominal wall incisions. The mouth of the bag was extracted from the umbilicus. Following this step, the optical trocar was introduced into the bag via the umbilical incision, and the bag was insufflated with CO_2_ to a pressure of 12 mmHg. The sleeves of the lower abdomen that were already outside the abdominal cavity were incised close to their tips. A 12- or 15-mm morcellator was introduced via the suprapubic incision, and a blunt grasper through the port sleeve in the left lower abdomen. Morcellation was carried out within the bag under direct vision. The instruments and the trocars in the lower abdomen were removed, the CO_2_ gas was released, and the port sleeves used during the operation were re-sealed using the closure strings provided by the manufacturer.

In addition to baseline data (age, body mass index, parity), the operating time for subtotal hysterectomy, the time needed to unfold the endobag and place the specimen in the bag, morcellation time, and complication rates were recorded for all procedures. The surgeons were asked to rate the level of difficulty in manipulating the bag (unfolding, sample positioning, morcellation, and bag removal) on a scale from 1 (easy) to 10 (extremely difficult).

### Statistical analysis

Statistical correlations were analyzed using Sigma Plot 12.3 for Windows (Systat Software Inc., Chicago, IL, USA). Spearman’s rank-order correlation was used to evaluate the association between the time taken to unfold the bag and prepare the sample for morcellation (bag preparation) and the level of difficulty on the one hand, and the remaining parameters on the other.

### Patient consent

The author(s) received and archived the patients’ consent to video recording/publication prior to recording the procedures.

## Results

### In vitro bag extraction

**Figure 5:**
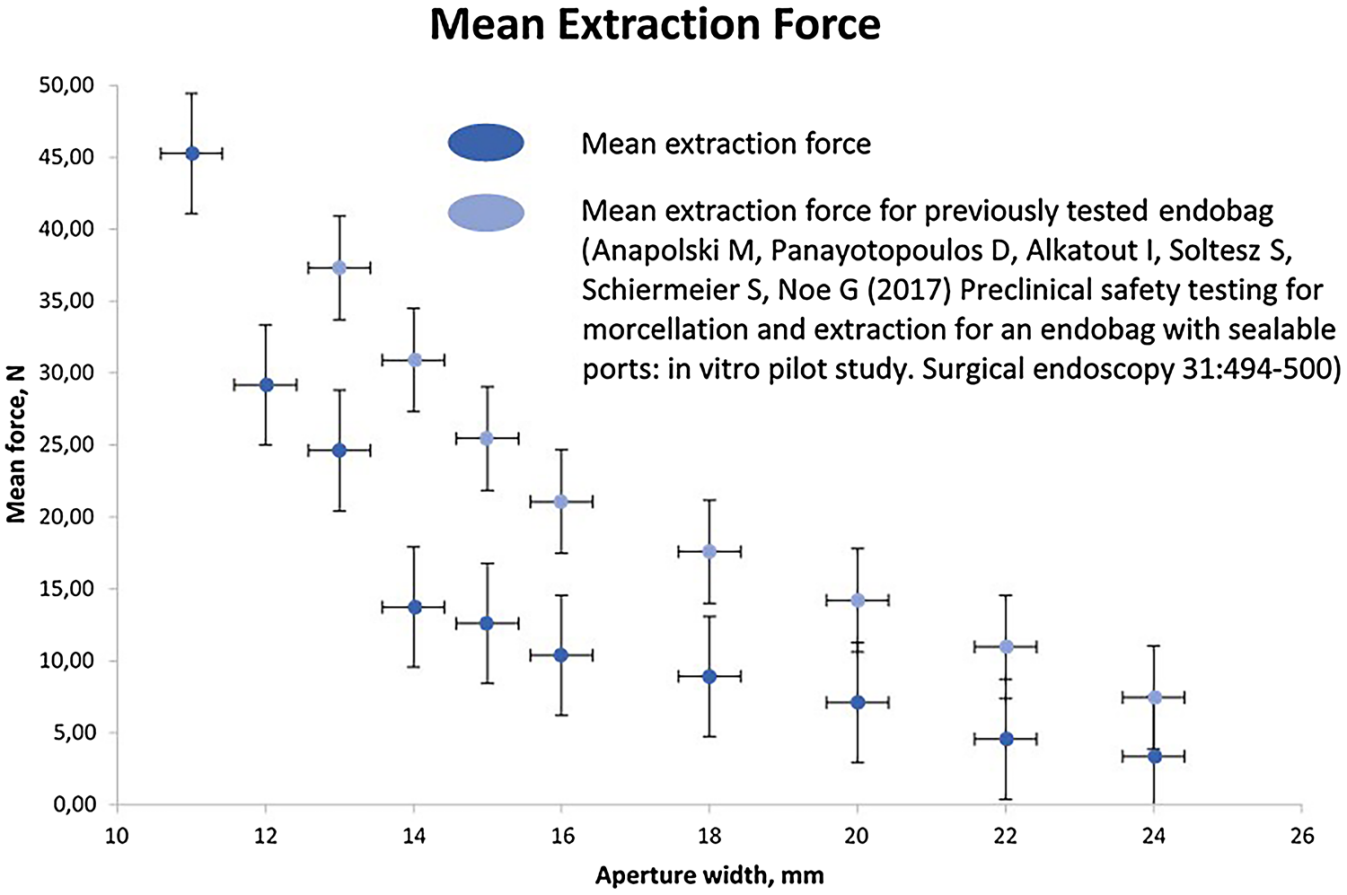
Force needed to extract the bag compared to a predecessor model.

Bag extraction could be performed through incisions measuring 11 to 24 mm in diameter. The bag could not be pulled through a 10-mm incision with reasonable force. No loss of blue dye—neither local nor remote—was registered for any incision (11 to 24 mm) during the experiement. The mean force required to extract the endobag is shown in Table 1. Figure 5 shows the relationship between aperture size and the mean extraction force compared to the previously tested endobag^21^.

**Table 1:** Aperture diameter and force needed to extract the bag through the template.

Aperture diameter (mm)	Mean extraction force (N)
24	3.43
22	4.41
20	6.37
18	8.33
16	12.74
15	13.72
14	12.75
13	23.05
12	30.40
11	47.56

### In-bag power morcellation

Fifty operations were performed by six surgeons. All of them had previous experience in performing in-bag morcellation and had conducted at least 24 procedures with other types of endobags. Twenty-six patients underwent a subtotal hysterectomy due to fibroids of hypermenorrhea, and 24 patients as a part of pelvic floor repair. Baseline characteristics of all patients are listed in Table 2. A 12-mm morcellator was used in 49 cases, and a 15-mm morcellator in one case. The mean operating time for subtotal hysterectomy was 53.4 min (range, 20–194 min). The mean bag preparation time was 7.1 min (range, 4–14 min), and the mean duration of the morcellation procedure 6.0 min (range, 1–39 min). After morcellation, the mean weight of residual tissue and fluid in the bag was 7.9 g (range, 0–39 g). The mean weight of the removed body of the uterus was 113.8 g (range, 13–896 g), and the mean level of difficulty 2.4 (range, 1–7).

**Table 2:** Baseline characteristics of patients.

Patients’ data	Mean (range)
Age	51.8 years (40–69)
Body mass index	25.5 (18.3–33.9)
Parity	1.72 (0–5)
**Indication for surgery (number of patients)**	
Prolapse surgery	26
Uterine bleeding disorders	15
Uterine myoma	9
Total number of patients	50

Linear regression analysis showed a significant influence (*p* < 0.05) of the duration of bag preparation on the overall duration of subtotal hysterectomy. Bag preparation time was significantly correlated with the patients’ body mass index (BMI) and the weight of uterine tissue (*p* < 0.05). The level of difficulty assigned by the surgeons was significantly correlated with BMI, the total duration of subtotal hysterectomy, blood loss, bag preparation time, the weight of residual tissue and fluid in the bag after morcellation, and the weight of uterine tissue (*p* < 0.05). The weight of tissue and fluid remaining in the bag after morcellation was also significantly correlated with BMI, the overall duration of subtotal hysterectomy, intraoperative blood loss, weight of uterine tissue, and the duration of morcellation (*p* < 0.05).

After the operation, three patients had a postoperative urinary tract infection that required antibiotic treatment. One patient had a persistent bleeding from the edge of the cervix that required a brief reoperation and coagulation; one patient had a postoperative hemorrhage after complex prolapse surgery, followed by a second laparoscopy and blood transfusion. No complications resulted from the use of the endobag.

## Discussion

Complications and difficulties associated with morcellation may be divided into two categories: those that can be reduced by optimization of clinical routine, and those that are native to the procedure itself. A systematic evaluation of morcellation-associated injuries will help to raise the awareness of surgeons and is also a crucial aspect of the clinical outcome. Milad and Milad reported that the vast majority of organ injuries were not described in the published literature, but were identified from the FDA database for the device^3,4^. Data collected after the introduction of power morcellation confirms that—like nearly all surgical devices—morcellators may cause life-threatening complications. Surgical experience and the ability to resolve the challenges of power morcellation are essential to reduce complication rates and treat potential injuries appropriately. Morcellation-related complication databases similar to the Manufacturer and User Facility Device Experience (MAUDE) of the FDA are useful to evaluate risks and minimize them in the future.

The widespread use of power morcellation for hysterectomy, fibroid surgery, renal tissue and other purposes has altered the requirements of histopathological investigation. Some reports in the published literature confirm that the pathologist may find it difficult to detect and classify changes in morcellated tissue^5,23^. Thus, the development of new operating techniques poses new challenges for all specialties involved in their use. Regrettably, the published literature provides no information about the effect of the diameter of the morcellator knife on the difficulties encountered in histopathological evaluation of morcellated tissue. The use of thin morcellators would result in greater tissue fragmentation. Reports of these difficulties would raise the awareness of the surgeon and improve the overall quality of medical treatment.

Whereas the previously described risks and difficulties cannot be fully eliminated, they can be significantly reduced by appropriate precautions such as lysis of adhesions and bowel mobilization. By contrast, dissemination of tissue and fluid within the abdominal cavity is an inherent element of power morcellation because of the need for multiple cuts and the presence of centripetal forces acting on the tissue. Spread of benign and malignant tissue in the abdomen may result in a series of medical conditions.

The overall risk of non-malignant changes after morcellation of the uterus or myoma is estimated at about 1%^24^. Benign conditions due to morcellation-related tissue dissemination are much more common than malignant changes. Benign conditions are usually less aggressive than malignant ones, but their presence might necessitate further surgery. Some authors suggest the use of containment bags in case morcellation is performed to extract resected tissue from the abdomen^24^.

Endometrial cancer accounts for more than 90% of all malignant diseases in the uterus. In a recently published study, a comparison of total abdominal hysterectomy and subtotal hysterectomy/laparoscopic myomectomy involving power morcellation revealed no significant difference in disease-specific mortality^25^. The authors suggested that the absence of a significant difference could be explained by the better prognosis of endometrial cancer compared to other uterine malignancies, and also the fact that the study data were derived from a limited geographical region^25^. The risk of accidental morcellation for endometrial cancer can be reduced by performing histological sampling (hysteroscopy and curettage) prior to surgery.

Smooth muscle tumors of uncertain malignant potential (STUMP) are rare histologic entities. Very little is known about their true incidence and long-term prognosis. These tumors bear a potential risk of malignant transformation and should be treated with caution^10,26^. Uterine sarcomas are rare, accounting for 3–7% of uterine neoplasms^14,15,27^. Following the case of a patient who experienced a dramatic course of disease after morcellation for a previously undiagnosed uterine malignancy, published in the Wall Street Journal^28^, the discussion concerning power morcellation became a highly emotional issue. An FDA warning issued in 2014 was based on the fact that a uterine sarcoma was diagnosed in 1 of 352 surgeries, and leiomyosarcoma in 1 of 498 surgeries for presumably benign fibroids^29^. Later the prevalence was shown to be 1 in 1428 surgeries, and thus much lower than the FDA estimate^16^. Whereas the meta-analysis performed by Pritts did not reveal a significant deterioration of the prognosis when the specimen was morcellated and not removed *en bloc*^16^, some authors have reported adverse effects of unprotected morcellation on overall survival^12,13^ and disease-free survival^12,30,31^.

Following the FDA warning, power morcellation was viewed with caution in many countries. According to recent surveys carried out in the USA and other countries, a large number of gynecologists stopped using power morcellation in order to avoid legal consequences^32–34^.

Most respondents of the above mentioned AAGL (American Association of Gynecologic Laparoscopists) and ACOG (American College of Obstetricians and Gynecologists) surveys (80.3%) believed that the FDA warning of 2014 did not improve patient outcomes^32^. This assumption is supported by Harris et al., who showed that minimally invasive hysterectomy was performed less frequently after the FDA safety communication, whereas major surgical non-transfusion-related complications and 30-day hospital readmission rates were increased^35^.

Based on prevalence rates for occult uterine malignancies and complication rates for surgical techniques, several statistical models have been proposed for a hypothetical cohort of patients. Compared to abdominal hysterectomy in patients with presumed fibroids, the laparoscopic approach was associated with a higher risk of accidental morcellation of uterine sarcoma. However, laparoscopy offered the advantage of a lower risk of hysterectomy-related deaths, lower rates of transfusion, surgical site infection, venous thromboembolism, incisional hernia, adhesion, and bowel obstruction. Laparoscopic hysterectomies would result in shorter hospital stays, earlier return to work, lesser need for postoperative analgesia, and better quality-of-life scores^36,37^. In summary, the currently available data suggest that the omission of power morcellation from the spectrum of surgery would signify a step backward for endoscopic surgery.

Although we lack reliable criteria to rule out malignancy prior to surgery, some decision algorithms have been proposed to select the most appropriate surgical access, depending on the individual patient’s risk profile. Siedhoff et al. referred to the high risk of unsuspected sarcoma in women above 50 years of age^38^. Based on the existing data, Sizzi et al. proposed a series of measures to reduce the risk of incidental morcellation of uterine sarcoma^39^. Further studies will be needed to establish validated risk assessment scores.

Irrigation of the peritoneal cavity after power morcellation may serve as a promising approach in terms of reducing the risk of tissue spread. Yu et al. demonstrated, in a pilot study, that irrigation with 3 L of saline after morcellation reduced the probability of detecting myoma cells after laparoscopic myomectomy^40^. Another important finding of the study was that myoma cells were detected even after myomectomy and before electromechanical morcellation. These data were supported by other authors, who reported significant cell spread during laparoscopic hysterectomies prior to the morcellation procedure^41^. This raises the question as to whether the risk of tissue spread can be attributed solely to electromechanical morcellation. The existing data show that the surgical steps performed before morcellation—such as preparation and dissection—bear the risk of dissemination, no matter how the tissue is removed from the abdominal cavity. Since sarcomas are known to spread via the blood stream, the risk of dissemination may be increased by transection of the tumor and not as a consequence of fragmentation.

Several endobag-based solutions were proposed for contained morcellation of uterine tissue^17–19,22,42^. The first endobags were developed as extraction bags. Additional features were added to reduce the risk of tissue and fluid loss^22^. It is assumed that the use of in-bag morcellation would reduce tissue dissemination in the abdominal cavity and thus diminish the risk of undesirable sequelae. Data acquired for T1 and T2 renal carcinoma suggest that manual in-bag morcellation resulted in similar survival rates as open nephrectomy, indicating the protective effect of contained morcellation^43^. Large-scale prospective randomized studies on the subject would hardly be compatible with general ethical principles. It would also be difficult to acquire, within a reasonable period of time, a large body of data concerning the reduction of risks associated with the use of contained morcellation. These aspects make it difficult to develop a suitable morcellation bag.

Tissue and fluid spread during power morcellation is usually caused by the rotating blade. In-bag morcellation was expected to reduce the risk of tissue dissemination secondary to the morcellation process. However, retrieval of the bag after morcellation was shown to be a crucial step. Forces applied on the bag to remove it from the abdomen might cause a loss of fluid or solid contents in this stage of surgery. A previously published in vitro study demonstrated that perforation of the bag during the morcellation process causes fluid leakage only during bag extraction^21^. Thus, a reliable closure mechanism for all openings of the endobag is crucial. Parameters for safe use of the bag—such as an incision of minimal diameter for bag retrieval—should be established before its clinical use. These parameters, which we determined under in vitro conditions, have been rarely addressed in the published literature concerning various types of morcellation bags. Where such data is provided, the authors mention that the safety of bag retrieval can be enhanced by enlarging the umbilical entry. In a previously published in vitro study, a minimum incision size of 18 mm was needed to achieve spillage-free endobag extraction from the abdomen^21^. In view of the fact that patients have described this incision site as the most painful compared to incisions for the insertion of lower abdominal trocars, it would be justified to conclude that patient safety was achieved at the cost of greater tissue trauma. The concept of routine dilatation of abdominal wall incisions for the very last step of the operation is, however, in direct contradiction to the principles of minimally invasive surgery.

Since the laparoscopic approach permits detailed inspection of the abdominal cavity, the retrieval of large tissue fragments during and after morcellation rarely is a major concern when using power morcellation. In contrast, very small tissue particles and fluid secondary to the morcellation procedure can hardly be extracted fully without the use of in-bag morcellation. Based on the results of the previously published in vitro study^21^ and our own unpublished data, we filled the bags only with stained fluid because the construction of closure mechanisms impermeable to fluid is more challenging than closure mechanisms that prevent the loss of solid content. Due to the design of the in vitro investigation, the present study yielded no information about the maximum size of solid specimens remaining in the bag that can be safely extracted through the template.

Compared to previous solutions, the endobag used in the present investigation offers a more reliable closure mechanism for trocar sleeves^19–21^. At the same time, the improved closure mechanism allows the surgeon to reduce the diameter of the umbilical incision. We demonstrated that the re-sealed bag can be removed safely via an 11-mm opening. This feature of the device would reduce the size of the umbilical incision. However, as the dynamic properties of the wooden template clearly differed from those of the abdominal wall, the results cannot be directly transferred to in vivo conditions. Moreover, it was impossible to consider variables as friction through the fascia, skin and subcutaneous tissues; these factors may modify the results under real-life conditions.

The data acquired in the present study show a direct correlation between bag preparation time, obesity, and the weight of the uterus. Increasing rates of obesity in many countries pose a serious problem for laparoscopy in general and the use of morcellation bags in particular. During the evolution of contained morcellation, some features were modified to improve ergonomics such as the introduction of pre-manufactured openings for two trocars in the lower abdomen^19,21^. Vargas et al. reported that in-bag morcellation required on average 26 min more than uncontained power morcellation^44^. The data from a pilot study based on an endobag with prefabricated closable incisions for two lower abdomen trocars showed that an additional 10.5 min were needed to insert the bag and prepare it for morcellation^20^. In the current study, we registered a mean bag preparation time of 7.1 min, which is considerably shorter than the time reported in previous studies^20,44^. However, it is difficult to compare these data because all surgeons of the present investigation had previous experience with other types of endobags. The use of in-bag procedures quite evidently takes more time. Prolonged surgery is also more costly. We noted that the time needed for bag preparation influenced the total duration of subtotal hysterectomy. Thus, the choice of the morcellation bag is important not only for the patient’s safety, but also in terms of the cost-effectiveness of the entire surgical procedure. As shown in the present study, the improvement of ergonomic parameters of containment bags may have a positive impact on operating time.

The device described in the present report offers the option of introducing three trocars in the lower abdomen. This would signify considerable freedom and comfort for surgeons who routinely work with three instrumental trocars. Since all trocar sleeves are sealed prior to use, the sleeves need to be re-sealed only if they are used for surgery. Thus, the surgeon is able to work with one, two or three ports in the lower abdomen, depending on uterus size, body mass index, and adhesions. Furthermore, it would dispense with the need for closure of unused sleeves and thus save time. Compared to the endobag described above^19–21^, the surgeon would save operating time for sleeve closure in case he/she is working with only one or two ports. As the use of containment bags is not reimbursed separately in the large majority of countries, the avoidance of surgical steps is an important economic factor.

We were able to unfold the endobag by applying the regular insufflation pressure of maximum 12 mmHg. In this respect, the endobag presented here is superior to some solutions described in the published literature, which involve the use of much greater CO_2_ pressure (25 mmHg) for this purpose^17^. On the one hand, such pressure levels are unphysiological and excessively high. On the other hand, the increased pressure within the bag might raise the risk of tissue or fluid loss^45^.

Unlike some solutions presented in the past, we were able to visualize abdominal organs outside the bag due to the transparency of the endobag material^17^. This is an important advantage in terms of the safety of the procedure. However, further comparative studies will be needed to confirm this notion.

Some authors describe the use of a special sheath for the optical device during morcellation in order to protect it against contamination^22^. We did not use a protective sheath because morcellation was the last step of the operation. This fact required a modification of some combined surgical procedures in terms of the sequence of individual surgical steps as well as the approach to anatomical structures^46,47^. Avoidance of the optical sleeve also reduces the cost of the containment bag.

Notably, the weight of residual tissue and fluid in the endobag after morcellation was significantly correlated with factors that make the intervention more complex: higher BMI, longer duration of surgery, higher blood loss, higher weight of the uterine sample, longer duration of the morcellation procedure, and a higher level of difficulty assigned by the surgeon. These results cannot be transferred to uncontained power morcellation. However, they show that the quantity of residual tissue and fluid in the abdominal cavity—and possibly the risk of dissemination of benign and malignant tissue—is not a constant value, but may be influenced by a number of objective and subjective factors.

## Conclusions

Taking into consideration the advantages of laparoscopic interventions compared to laparotomy, the total abandonment of surgical procedures such as fibroid removal or subtotal hysterectomy would signify a step back in modern surgery. Several measures could be used to reduce the risk of dissemination. However, it would be difficult to evaluate the efficacy of these measures since such evaluation would involve second-look surgeries.

Preoperative risk assessment scales should be used to reduce the likelihood of accidental morcellation of previously unknown uterine malignancies^38,39^. Irrigation of the peritoneal cavity with saline was shown to reduce the quantity of residual tissue after the morcellation procedure^40^. The use of containment bags could reduce the risk of tissue dissemination and fluid spillage attributed to the morcellation process, but further evidence will be needed to confirm this thesis. Operating times and costs must be monitored and considered in relation to the respective reimbursement system.

The additional time taken to prepare the bag for morcellation was shorter than the times reported in the published literature for similar technical solutions^20,44^. In contrast to uncontained morcellation, which involves the removal of tissue fragments and potential spillage of these into the abdominal cavity, the morcellation bag involves no such steps and could well shorten the final phase of surgery.

We believe that the device described in the present study could enhance the safety of in-bag morcellation and simultaneously reduce tissue trauma by reducing the size of the umbilical incision, but further data will be needed to confirm this statement. The introduction of three sealed and re-sealable ports in addition to the mouth of the bag permits adaptation of the bag to individual needs.

## Supplementary Information

Supplementary Information 1.
